# La langue noire villeuse: après chimiothérapie

**DOI:** 10.11604/pamj.2014.18.69.4574

**Published:** 2014-05-22

**Authors:** Sihame Lkhoyaali, Hassan Errihani

**Affiliations:** 1 Department of Medical Oncology, National Institute of Oncology, Rabat, Morocco

**Keywords:** Langue noire villeuse, chimiothérapie, CHOP, Lingua villosa nigra, chimiotherapy, CHOP

## Image en medicine

Nous rapportons le cas d'une patiente âgée de 70 ans, non tabagique hypertendue sous inhibiteur calcique, suivie pour un lymphome à grandes cellules B orbitaire ayant reçu une chimiothérapie à base de CHOP (cyclophosphamide, adriamycine, vincristine, et prednisone) ayant accusé après 2 cures à j15 de la 2ème cure de chimiothérapie, des douleurs au niveau de la langue, l'examen clinique trouve une coloration noirâtre au niveau de la langue correspondant à la langue noire villeuse; un écouvillonnage de la langue et de la gorge ont été réalisés, le test de l'antigène streptococcique rapide ainsi que la culture étaient négatifs. Après un traitement à base de bains de bouche fait de bicarbonate de sodium l’évolution a été marquée par la disparition de la coloration noirâtre au bout de 4 semaines. La langue noire villeuse (Lingua Villosa nigra) correspond à un revêtement noir anormal sur la surface dorsale de la langue, les côtés et l'extrémité de la langue sont rarement impliqués, elle est causée par la desquamation défectueuse de la langue avec l'allongement et l'hypertrophie de la papille filiforme souvent liée à une infection par des germes ou le plus souvent par le candida albicans. Il s'agit d'une pathologie bénigne et spontanément résolutive.

**Figure 1:**
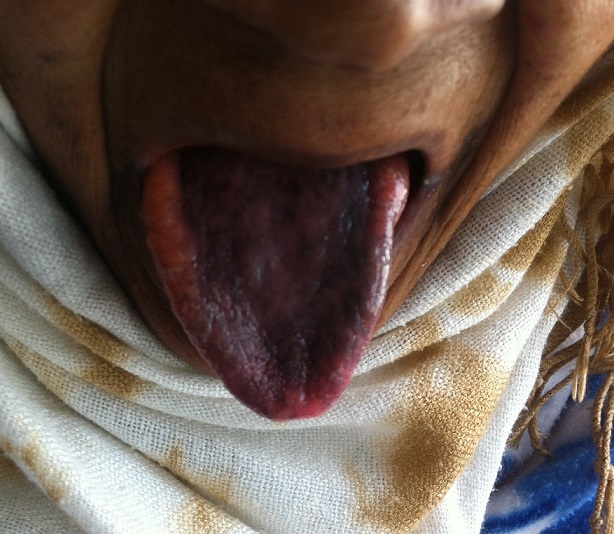
Aspect noiratre de la partie centrale de langue correspondant à la “langue noire villeuse”

